# Image collection of 3D-printed prototypes and non-3D-printed prototypes

**DOI:** 10.1016/j.dib.2019.104691

**Published:** 2019-10-29

**Authors:** Matthew Li, Ninad Mahajan, Jessica Menold, Christopher McComb

**Affiliations:** Pennsylvania State University, USA

**Keywords:** Image classification, Prototypes, Engineering design, Machine learning, Deep learning

## Abstract

Image processing refers to the use of computer algorithms to manipulate and enhance digital images to improve their quality or to make them more suitable for tasks such as classification. Common benchmarking datasets in this field include the imagenet, CIFAR-100, and MNIST datasets. This dataset is a collection of images that are particularly relevant to engineering and design, consisting of two categories: 3D-printed prototypes, and non-3D-printed prototypes This data was collected through a hybrid approach that entailed both web scraping and direct collection from engineering labs and workspaces at Penn State University. The initial data was then augmented using several data augmentation techniques including rotation, noise, blur, and color shifting. This dataset is potentially useful to train image classification algorithms or attentional mapping approaches. This data can be used either by itself or used to bolster an existing image classification dataset.

**Table:** Specifications table

Subject	Engineering
Specific subject area	Training data for image classification
Type of data	Images
How data were acquired	Smartphone photography, web scraping, enhanced with OpenCV
Data format	processed image data (JPEG)
Parameters for data collection	clear photographs of real engineering prototypes were considered for collection
Description of data collection	images of 3d-printed vs non-3d-printed prototypes
Data source location	Pennsylvania State University University Park, Pennsylvania USA
Data accessibility	With the article

**Table:** 

**Value of the Data** • This data can be used as a standalone dataset or to supplement existing image datasets • This data may be of particular use to individuals interested in prototyping practices, characteristics, and features • This data can be used for experimentation in neural network training for image processing tasks

## 1 Data

**Fig. 1:**
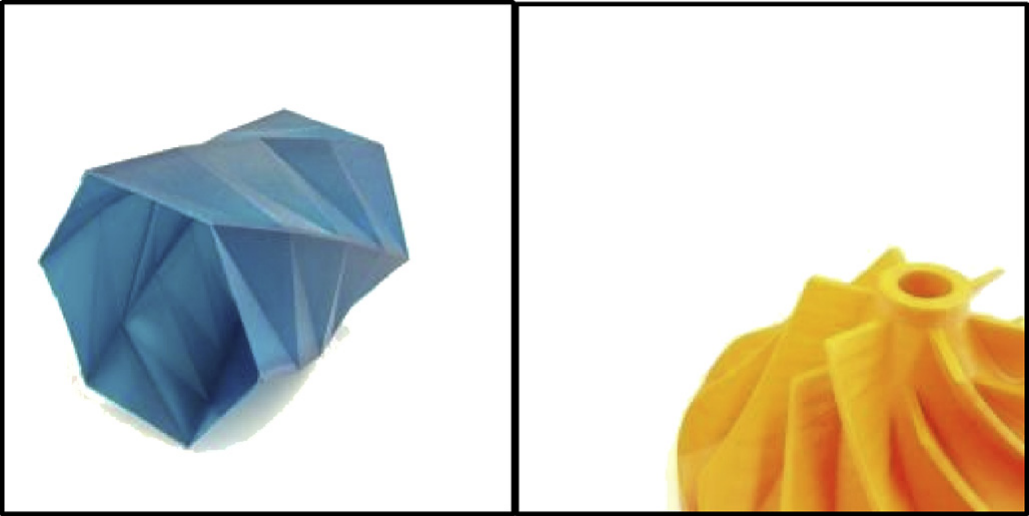
3D-printed prototypes.

**Fig. 2:**
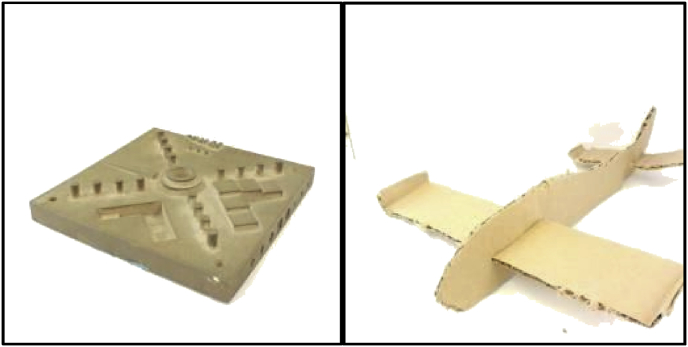
Non-3D-printed prototypes.

This dataset consists of images with a resolution of 256 × 256 pixels, split into two classes: “3D-printed prototypes” and “non-3D-printed prototypes.” There are a total of 51,520 images, with 25,760 images in each category. Examples of the 3D-printed prototypes are provided in Fig. 1, and examples of non-3D-printed prototypes in Fig. 2.

## 2 Experimental design, materials, and methods

Half of the images in the dataset were taken by the researchers themselves for the purpose of this study. Penn State University's engineering classrooms are home to many 3D-printers, which meant ample amounts of 3D-printed models were available to be photographed as data. We constructed small lightboxes using cardboard and white paper, and photographed a wide selection of 3D-printed models at various angles and orientations using smartphones. By modifying the exposure and white balance, we were able to get photographs of every object with a pure white background, with nothing but the object in frame (1). The same was done for a selection of non-3D-printed objects found in the same setting (2). These prototypes were produced using cardboard, metal, and other low-fidelity materials. 805 images were gathered in this way.

The rest of the images were gathered from google images using a simple web-scraping script written in python. Searching for 3D-printed models was straightforward, but because “non-3D-printed object” is such a blanket term, the search for this category was limited to engineering prototypes only. Both these methods resulted in a total of 1610 images, with 805 in each category. A uniform aspect ratio and resolution were achieved by reshaping the images using image processing tools in OpenCV.

**Fig. 3:**
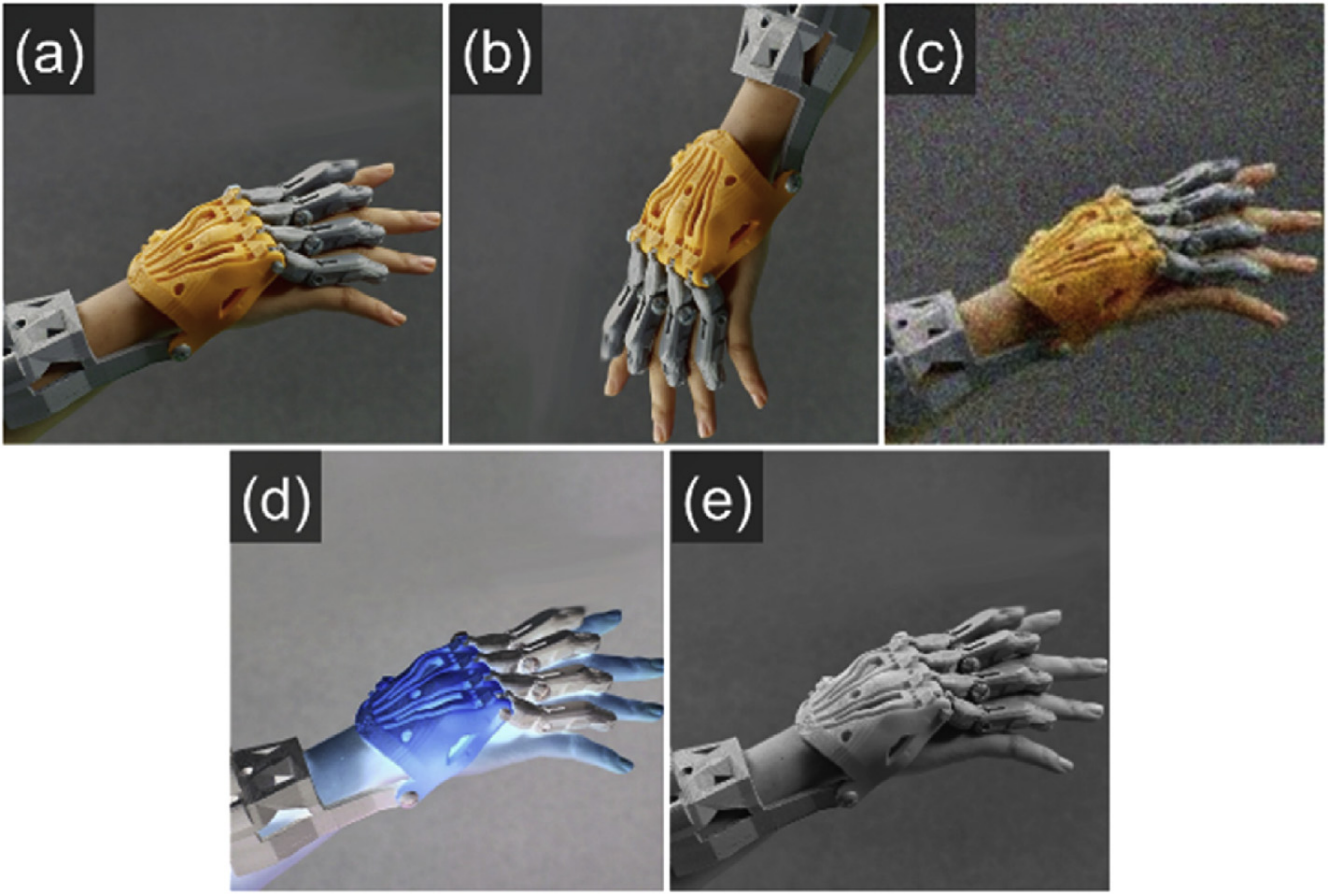
(a) original image; (b) rotation and reflection; (c) addition of Gaussian noise; (d) color negation; (e) grayscale conversion.

This problem was addressed through data augmentation. A series of common image manipulation techniques were used--each image was rotated 90, 180, and 270°, resulting in four images. Each of the four images were then flipped (reversed) (see Fig. 3b), resulting in eight. Each of the eight were then treated to gaussian noise (see Fig. 3c), color negation (see Fig. 3d), and grayscale conversion (see Fig. 3e). Including the unaltered original image, this results in 32 images, expanding our original dataset of 1610 images by a factor of 32. This finalized dataset comprised of 51,520 images, with 25,760 in each category. This data augmentation was performed using tools in the OpenCV library in a simple python script.

