# Supplementary figures and images for: Image collection of 3D-printed prototypes and non-3D-printed prototypes (part 2 of 2)
Source: Data Brief. 2019 Oct 29;27:104691. doi: 10.1016/j.dib.2019.104691 (PMC6920504; doi:10.1016/j.dib.2019.104691)

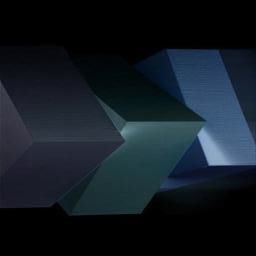

Supplement: Multimedia component 1 [file mmc1.zip › images/not_3d_printed/023947.jpg]

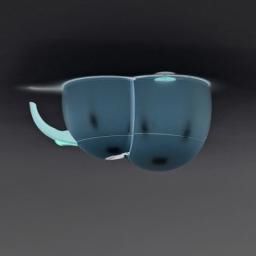

Supplement: Multimedia component 1 [file mmc1.zip › images/not_3d_printed/002989.jpg]

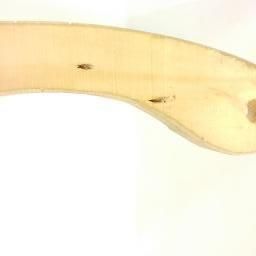

Supplement: Multimedia component 1 [file mmc1.zip › images/not_3d_printed/004320.jpg]

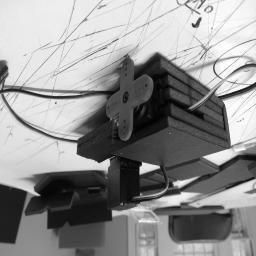

Supplement: Multimedia component 1 [file mmc1.zip › images/not_3d_printed/014133.jpg]

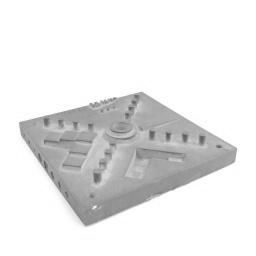

Supplement: Multimedia component 1 [file mmc1.zip › images/not_3d_printed/003329.jpg]

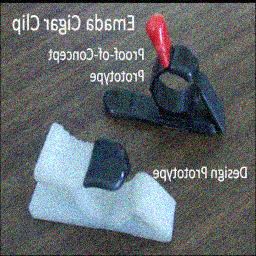

Supplement: Multimedia component 1 [file mmc1.zip › images/not_3d_printed/005980.jpg]

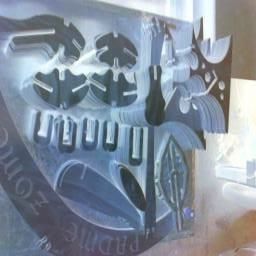

Supplement: Multimedia component 1 [file mmc1.zip › images/not_3d_printed/014655.jpg]

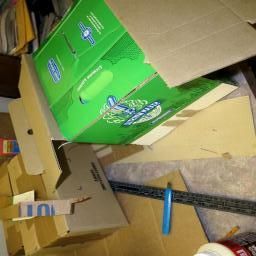

Supplement: Multimedia component 1 [file mmc1.zip › images/not_3d_printed/024796.jpg]

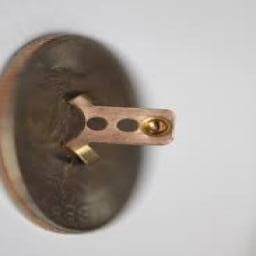

Supplement: Multimedia component 1 [file mmc1.zip › images/not_3d_printed/004446.jpg]

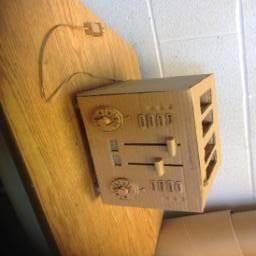

Supplement: Multimedia component 1 [file mmc1.zip › images/not_3d_printed/012224.jpg]

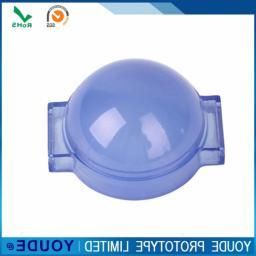

Supplement: Multimedia component 1 [file mmc1.zip › images/not_3d_printed/025488.jpg]

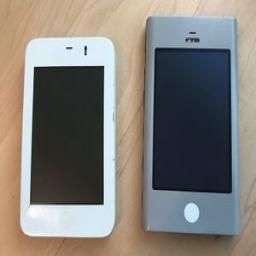

Supplement: Multimedia component 1 [file mmc1.zip › images/not_3d_printed/005758.jpg]

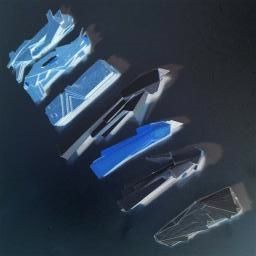

Supplement: Multimedia component 1 [file mmc1.zip › images/not_3d_printed/002037.jpg]

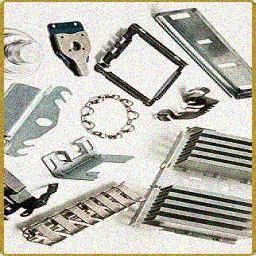

Supplement: Multimedia component 1 [file mmc1.zip › images/not_3d_printed/016042.jpg]

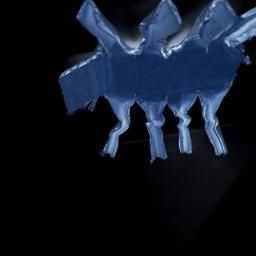

Supplement: Multimedia component 1 [file mmc1.zip › images/not_3d_printed/019371.jpg]

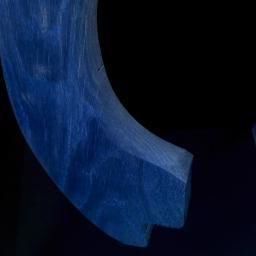

Supplement: Multimedia component 1 [file mmc1.zip › images/not_3d_printed/006251.jpg]

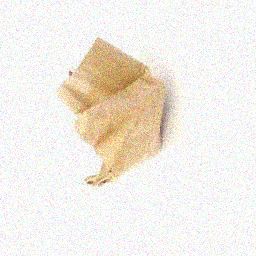

Supplement: Multimedia component 1 [file mmc1.zip › images/not_3d_printed/009162.jpg]

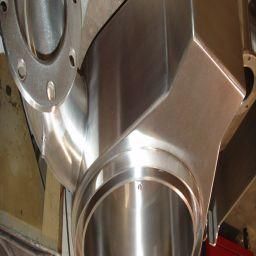

Supplement: Multimedia component 1 [file mmc1.zip › images/not_3d_printed/021836.jpg]

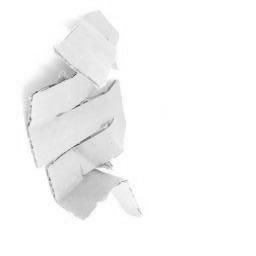

Supplement: Multimedia component 1 [file mmc1.zip › images/not_3d_printed/010433.jpg]

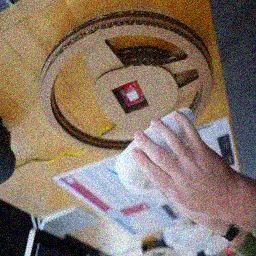

Supplement: Multimedia component 1 [file mmc1.zip › images/not_3d_printed/000620.jpg]

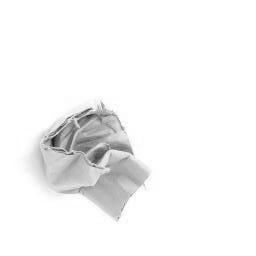

Supplement: Multimedia component 1 [file mmc1.zip › images/not_3d_printed/008297.jpg]

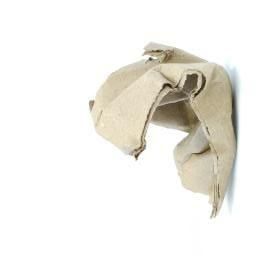

Supplement: Multimedia component 1 [file mmc1.zip › images/not_3d_printed/018084.jpg]

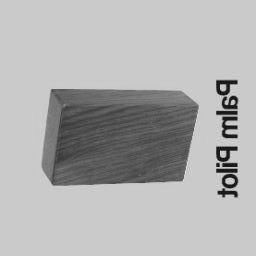

Supplement: Multimedia component 1 [file mmc1.zip › images/not_3d_printed/021605.jpg]

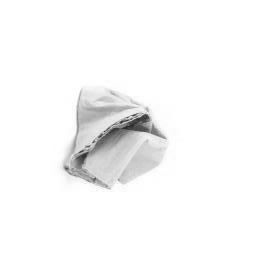

Supplement: Multimedia component 1 [file mmc1.zip › images/not_3d_printed/009189.jpg]

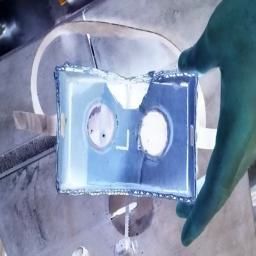

Supplement: Multimedia component 1 [file mmc1.zip › images/not_3d_printed/000813.jpg]

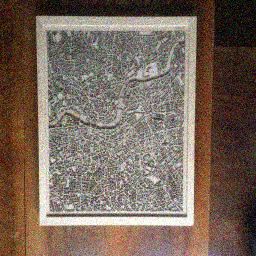

Supplement: Multimedia component 1 [file mmc1.zip › images/not_3d_printed/014866.jpg]

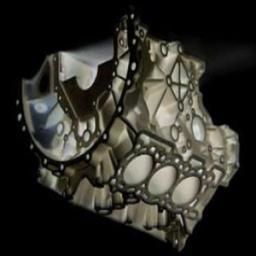

Supplement: Multimedia component 1 [file mmc1.zip › images/not_3d_printed/025463.jpg]

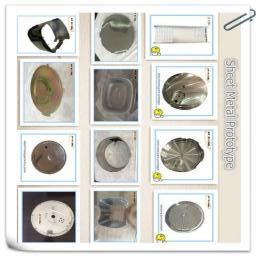

Supplement: Multimedia component 1 [file mmc1.zip › images/not_3d_printed/023012.jpg]

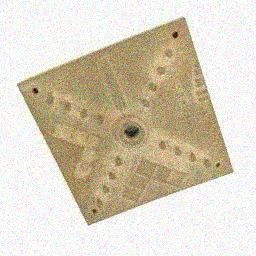

Supplement: Multimedia component 1 [file mmc1.zip › images/not_3d_printed/002962.jpg]

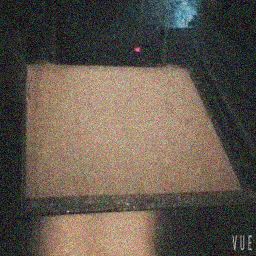

Supplement: Multimedia component 1 [file mmc1.zip › images/not_3d_printed/023774.jpg]

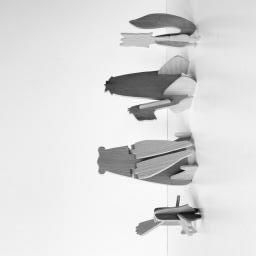

Supplement: Multimedia component 1 [file mmc1.zip › images/not_3d_printed/025305.jpg]

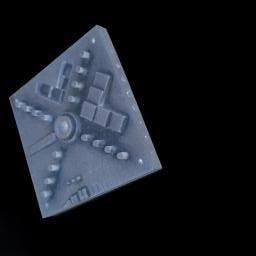

Supplement: Multimedia component 1 [file mmc1.zip › images/not_3d_printed/021163.jpg]

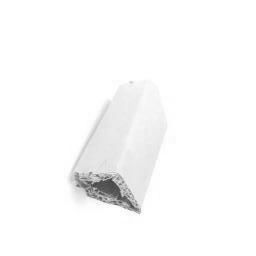

Supplement: Multimedia component 1 [file mmc1.zip › images/not_3d_printed/009837.jpg]

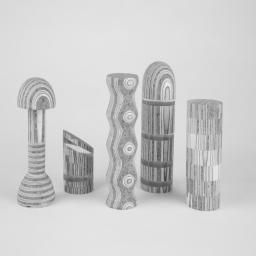

Supplement: Multimedia component 1 [file mmc1.zip › images/not_3d_printed/016917.jpg]

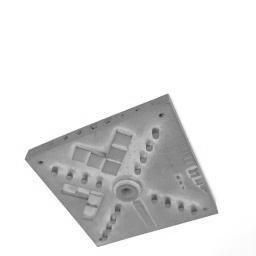

Supplement: Multimedia component 1 [file mmc1.zip › images/not_3d_printed/021177.jpg]

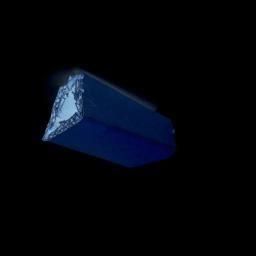

Supplement: Multimedia component 1 [file mmc1.zip › images/not_3d_printed/009823.jpg]

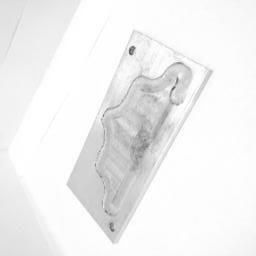

Supplement: Multimedia component 1 [file mmc1.zip › images/not_3d_printed/020269.jpg]

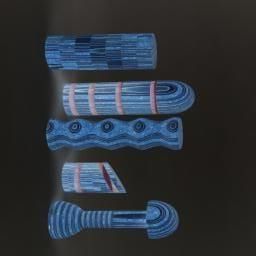

Supplement: Multimedia component 1 [file mmc1.zip › images/not_3d_printed/016903.jpg]

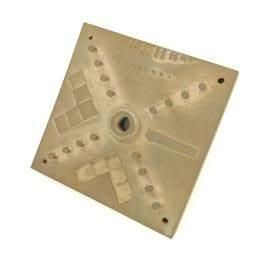

Supplement: Multimedia component 1 [file mmc1.zip › images/not_3d_printed/002976.jpg]

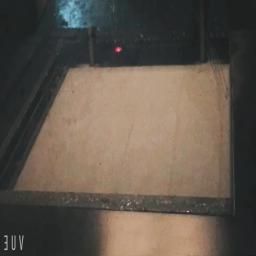

Supplement: Multimedia component 1 [file mmc1.zip › images/not_3d_printed/023760.jpg]

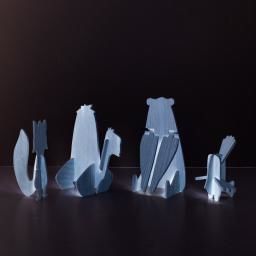

Supplement: Multimedia component 1 [file mmc1.zip › images/not_3d_printed/025311.jpg]

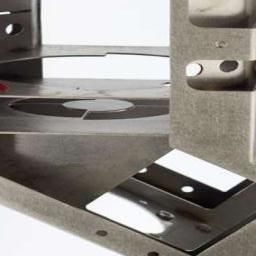

Supplement: Multimedia component 1 [file mmc1.zip › images/not_3d_printed/014872.jpg]

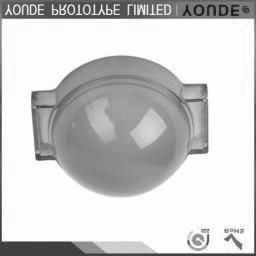

Supplement: Multimedia component 1 [file mmc1.zip › images/not_3d_printed/025477.jpg]

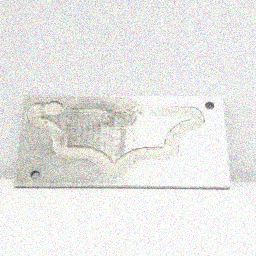

Supplement: Multimedia component 1 [file mmc1.zip › images/not_3d_printed/022318.jpg]

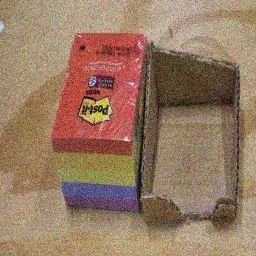

Supplement: Multimedia component 1 [file mmc1.zip › images/not_3d_printed/023006.jpg]

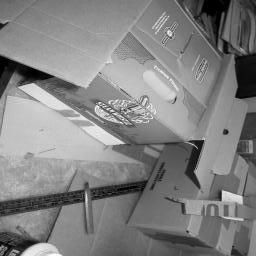

Supplement: Multimedia component 1 [file mmc1.zip › images/not_3d_printed/024769.jpg]

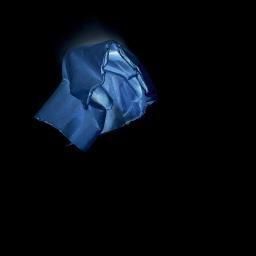

Supplement: Multimedia component 1 [file mmc1.zip › images/not_3d_printed/008283.jpg]

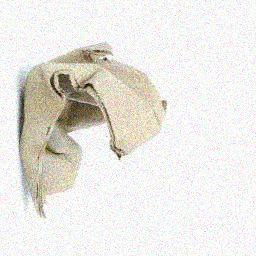

Supplement: Multimedia component 1 [file mmc1.zip › images/not_3d_printed/018090.jpg]

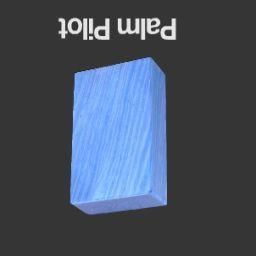

Supplement: Multimedia component 1 [file mmc1.zip › images/not_3d_printed/021611.jpg]

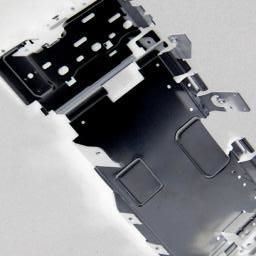

Supplement: Multimedia component 1 [file mmc1.zip › images/not_3d_printed/000807.jpg]

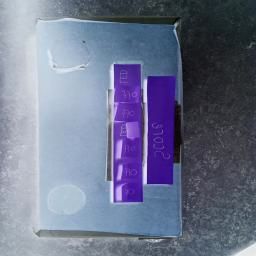

Supplement: Multimedia component 1 [file mmc1.zip › images/not_3d_printed/024999.jpg]

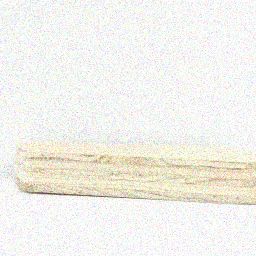

Supplement: Multimedia component 1 [file mmc1.zip › images/not_3d_printed/022330.jpg]

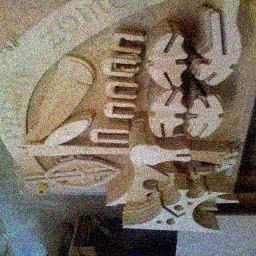

Supplement: Multimedia component 1 [file mmc1.zip › images/not_3d_printed/014682.jpg]

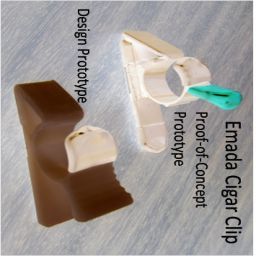

Supplement: Multimedia component 1 [file mmc1.zip › images/not_3d_printed/005957.jpg]

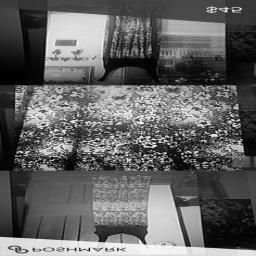

Supplement: Multimedia component 1 [file mmc1.zip › images/not_3d_printed/024741.jpg]

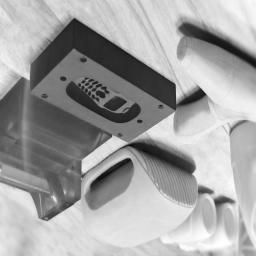

Supplement: Multimedia component 1 [file mmc1.zip › images/not_3d_printed/004491.jpg]

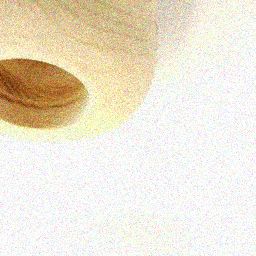

Supplement: Multimedia component 1 [file mmc1.zip › images/not_3d_printed/007198.jpg]

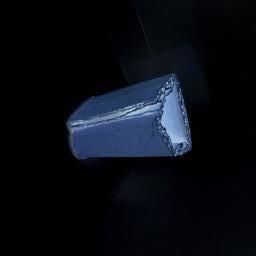

Supplement: Multimedia component 1 [file mmc1.zip › images/not_3d_printed/020527.jpg]

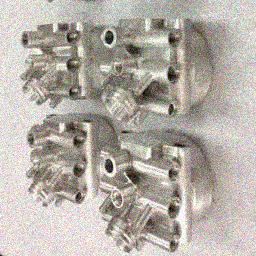

Supplement: Multimedia component 1 [file mmc1.zip › images/not_3d_printed/011922.jpg]

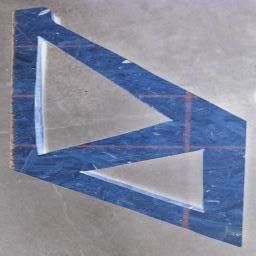

Supplement: Multimedia component 1 [file mmc1.zip › images/not_3d_printed/016095.jpg]

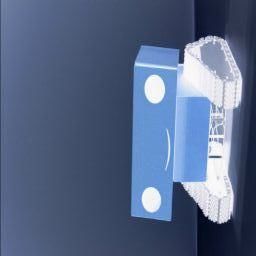

Supplement: Multimedia component 1 [file mmc1.zip › images/not_3d_printed/021639.jpg]

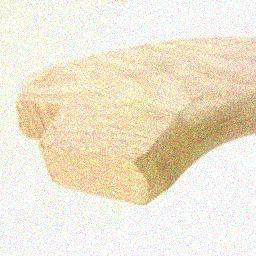

Supplement: Multimedia component 1 [file mmc1.zip › images/not_3d_printed/006286.jpg]

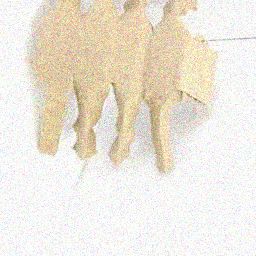

Supplement: Multimedia component 1 [file mmc1.zip › images/not_3d_printed/018906.jpg]

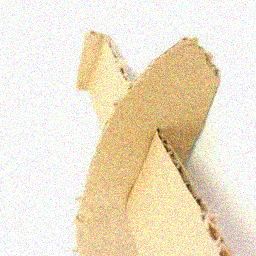

Supplement: Multimedia component 1 [file mmc1.zip › images/not_3d_printed/007826.jpg]

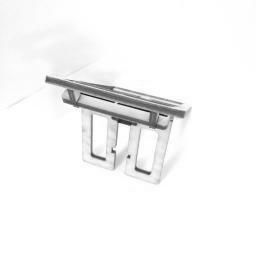

Supplement: Multimedia component 1 [file mmc1.zip › images/not_3d_printed/020241.jpg]

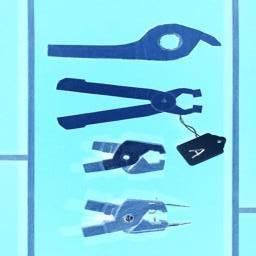

Supplement: Multimedia component 1 [file mmc1.zip › images/not_3d_printed/000191.jpg]

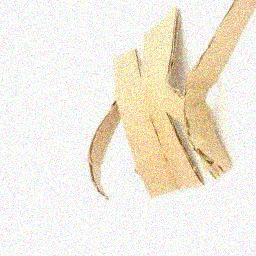

Supplement: Multimedia component 1 [file mmc1.zip › images/not_3d_printed/010382.jpg]

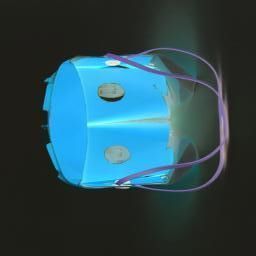

Supplement: Multimedia component 1 [file mmc1.zip › images/not_3d_printed/024027.jpg]

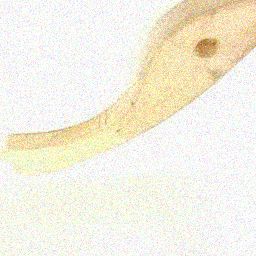

Supplement: Multimedia component 1 [file mmc1.zip › images/not_3d_printed/003498.jpg]

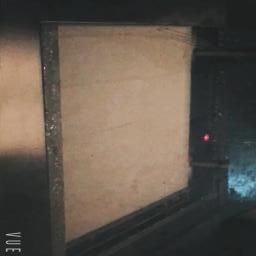

Supplement: Multimedia component 1 [file mmc1.zip › images/not_3d_printed/023748.jpg]

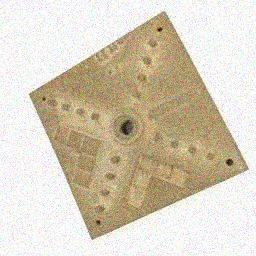

Supplement: Multimedia component 1 [file mmc1.zip › images/not_3d_printed/002786.jpg]

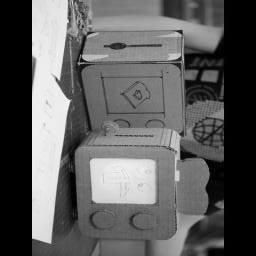

Supplement: Multimedia component 1 [file mmc1.zip › images/not_3d_printed/013853.jpg]

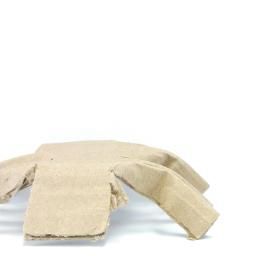

Supplement: Multimedia component 1 [file mmc1.zip › images/not_3d_printed/022456.jpg]

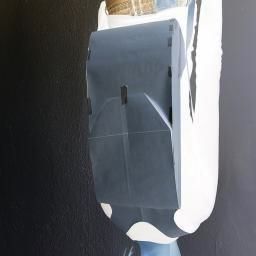

Supplement: Multimedia component 1 [file mmc1.zip › images/not_3d_printed/012595.jpg]

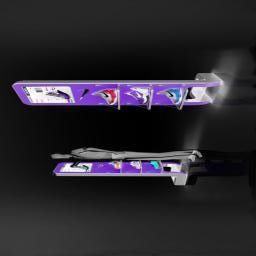

Supplement: Multimedia component 1 [file mmc1.zip › images/not_3d_printed/025339.jpg]

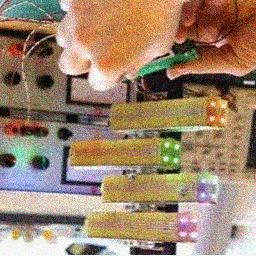

Supplement: Multimedia component 1 [file mmc1.zip › images/not_3d_printed/023990.jpg]

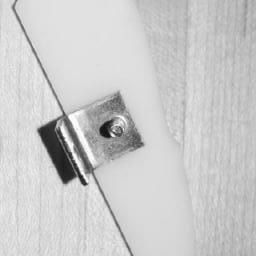

Supplement: Multimedia component 1 [file mmc1.zip › images/not_3d_printed/024033.jpg]

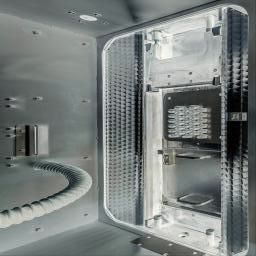

Supplement: Multimedia component 1 [file mmc1.zip › images/not_3d_printed/013847.jpg]

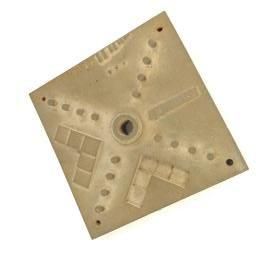

Supplement: Multimedia component 1 [file mmc1.zip › images/not_3d_printed/002792.jpg]

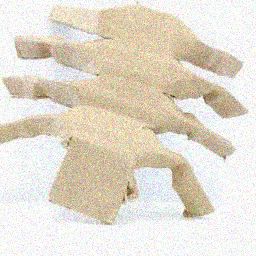

Supplement: Multimedia component 1 [file mmc1.zip › images/not_3d_printed/022442.jpg]

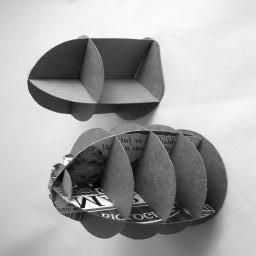

Supplement: Multimedia component 1 [file mmc1.zip › images/not_3d_printed/012581.jpg]

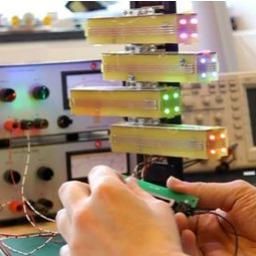

Supplement: Multimedia component 1 [file mmc1.zip › images/not_3d_printed/023984.jpg]

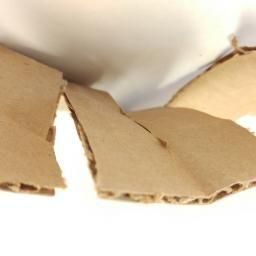

Supplement: Multimedia component 1 [file mmc1.zip › images/not_3d_printed/011088.jpg]

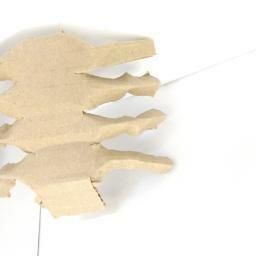

Supplement: Multimedia component 1 [file mmc1.zip › images/not_3d_printed/018912.jpg]

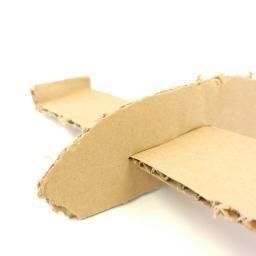

Supplement: Multimedia component 1 [file mmc1.zip › images/not_3d_printed/007832.jpg]

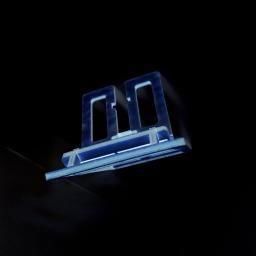

Supplement: Multimedia component 1 [file mmc1.zip › images/not_3d_printed/020255.jpg]

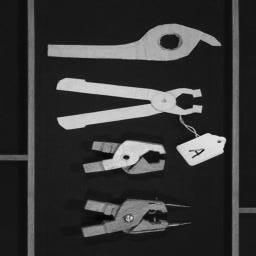

Supplement: Multimedia component 1 [file mmc1.zip › images/not_3d_printed/000185.jpg]

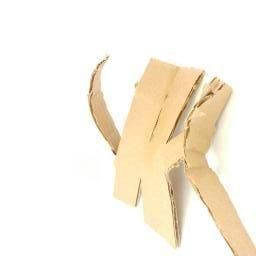

Supplement: Multimedia component 1 [file mmc1.zip › images/not_3d_printed/010396.jpg]

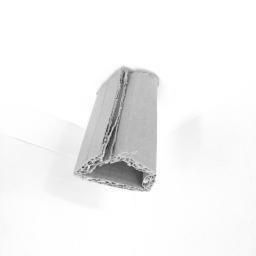

Supplement: Multimedia component 1 [file mmc1.zip › images/not_3d_printed/020533.jpg]

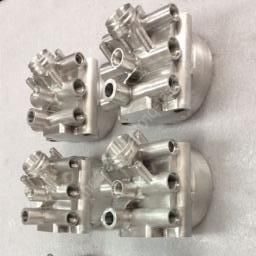

Supplement: Multimedia component 1 [file mmc1.zip › images/not_3d_printed/011936.jpg]

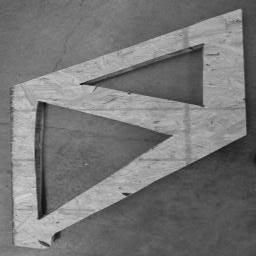

Supplement: Multimedia component 1 [file mmc1.zip › images/not_3d_printed/016081.jpg]

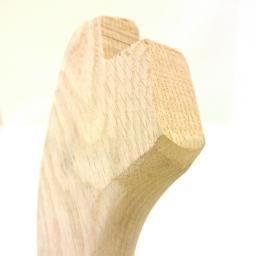

Supplement: Multimedia component 1 [file mmc1.zip › images/not_3d_printed/006292.jpg]

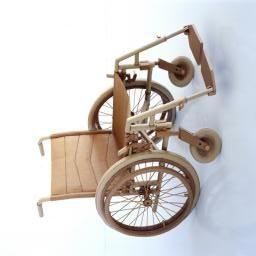

Supplement: Multimedia component 1 [file mmc1.zip › images/not_3d_printed/015588.jpg]

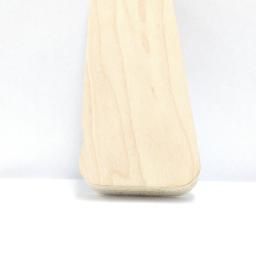

Supplement: Multimedia component 1 [file mmc1.zip › images/not_3d_printed/022324.jpg]

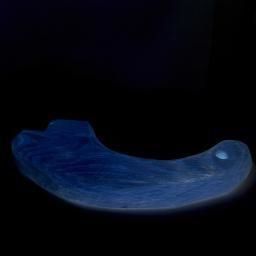

Supplement: Multimedia component 1 [file mmc1.zip › images/not_3d_printed/005943.jpg]

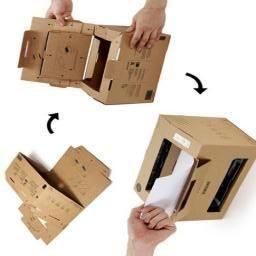

Supplement: Multimedia component 1 [file mmc1.zip › images/not_3d_printed/014696.jpg]

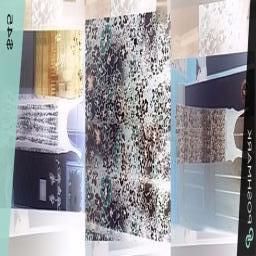

Supplement: Multimedia component 1 [file mmc1.zip › images/not_3d_printed/024755.jpg]

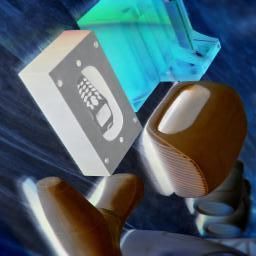

Supplement: Multimedia component 1 [file mmc1.zip › images/not_3d_printed/004485.jpg]

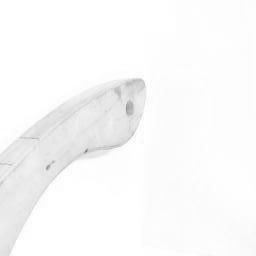

Supplement: Multimedia component 1 [file mmc1.zip › images/not_3d_printed/003665.jpg]

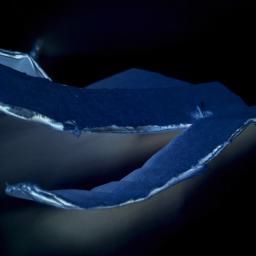

Supplement: Multimedia component 1 [file mmc1.zip › images/not_3d_printed/014319.jpg]
