# Supplementary figures and images for: Image collection of 3D-printed prototypes and non-3D-printed prototypes (part 1 of 2)
Source: Data Brief. 2019 Oct 29;27:104691. doi: 10.1016/j.dib.2019.104691 (PMC6920504; doi:10.1016/j.dib.2019.104691)

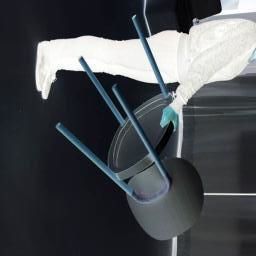

Supplement: Multimedia component 1 [file mmc1.zip › images/not_3d_printed/003301.jpg]

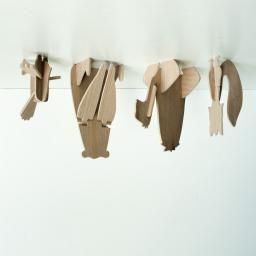

Supplement: Multimedia component 1 [file mmc1.zip › images/not_3d_printed/013112.jpg]

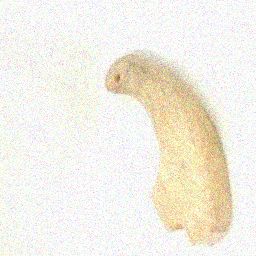

Supplement: Multimedia component 1 [file mmc1.zip › images/not_3d_printed/005770.jpg]

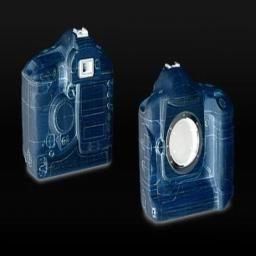

Supplement: Multimedia component 1 [file mmc1.zip › images/not_3d_printed/015563.jpg]

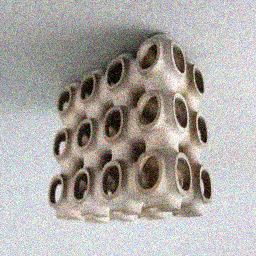

Supplement: Multimedia component 1 [file mmc1.zip › images/not_3d_printed/024966.jpg]

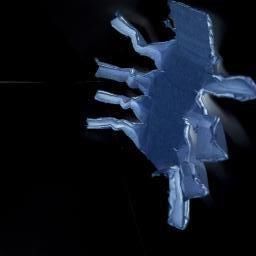

Supplement: Multimedia component 1 [file mmc1.zip › images/not_3d_printed/019359.jpg]

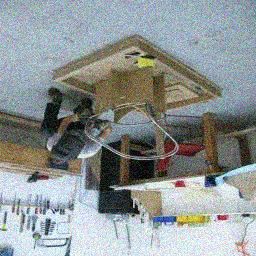

Supplement: Multimedia component 1 [file mmc1.zip › images/not_3d_printed/001516.jpg]

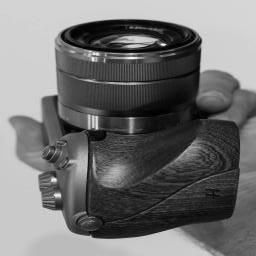

Supplement: Multimedia component 1 [file mmc1.zip › images/not_3d_printed/011705.jpg]

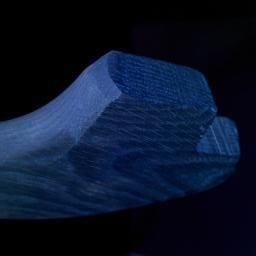

Supplement: Multimedia component 1 [file mmc1.zip › images/not_3d_printed/006279.jpg]

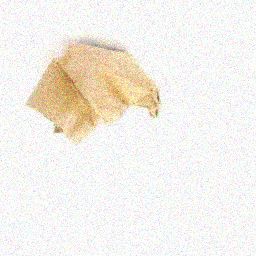

Supplement: Multimedia component 1 [file mmc1.zip › images/not_3d_printed/008254.jpg]

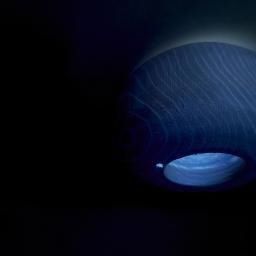

Supplement: Multimedia component 1 [file mmc1.zip › images/not_3d_printed/007167.jpg]

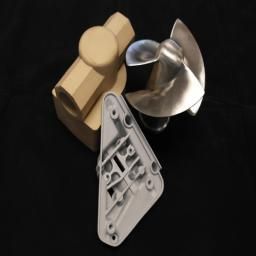

Supplement: Multimedia component 1 [file mmc1.zip › images/not_3d_printed/000608.jpg]

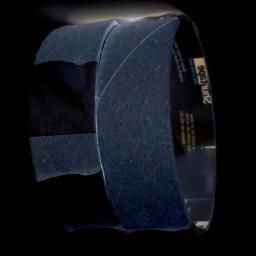

Supplement: Multimedia component 1 [file mmc1.zip › images/not_3d_printed/018047.jpg]

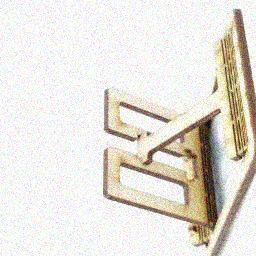

Supplement: Multimedia component 1 [file mmc1.zip › images/not_3d_printed/017374.jpg]

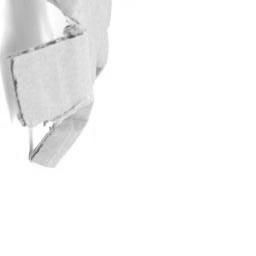

Supplement: Multimedia component 1 [file mmc1.zip › images/not_3d_printed/018721.jpg]

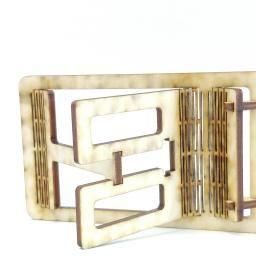

Supplement: Multimedia component 1 [file mmc1.zip › images/not_3d_printed/017412.jpg]

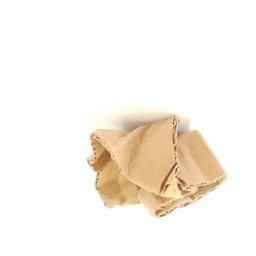

Supplement: Multimedia component 1 [file mmc1.zip › images/not_3d_printed/008532.jpg]

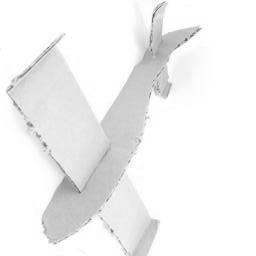

Supplement: Multimedia component 1 [file mmc1.zip › images/not_3d_printed/007601.jpg]

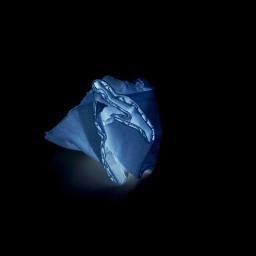

Supplement: Multimedia component 1 [file mmc1.zip › images/not_3d_printed/011063.jpg]

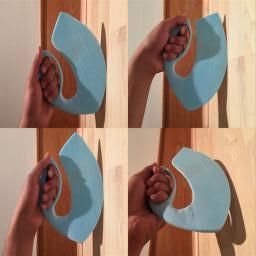

Supplement: Multimedia component 1 [file mmc1.zip › images/not_3d_printed/001270.jpg]

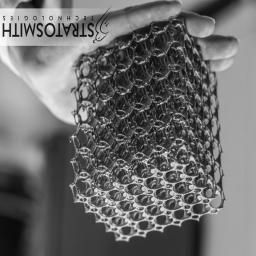

Supplement: Multimedia component 1 [file mmc1.zip › images/not_3d_printed/015205.jpg]

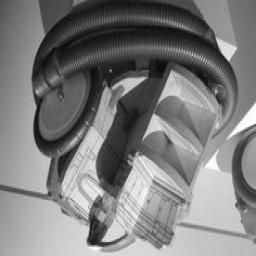

Supplement: Multimedia component 1 [file mmc1.zip › images/not_3d_printed/002779.jpg]

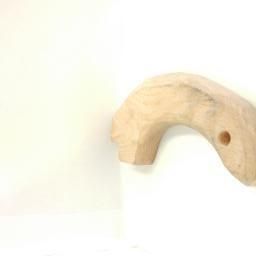

Supplement: Multimedia component 1 [file mmc1.zip › images/not_3d_printed/005016.jpg]

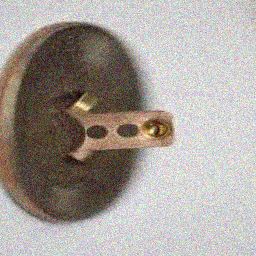

Supplement: Multimedia component 1 [file mmc1.zip › images/not_3d_printed/004308.jpg]

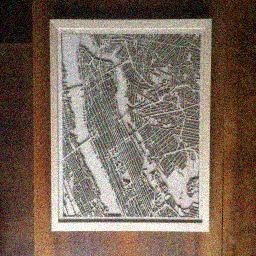

Supplement: Multimedia component 1 [file mmc1.zip › images/not_3d_printed/013674.jpg]

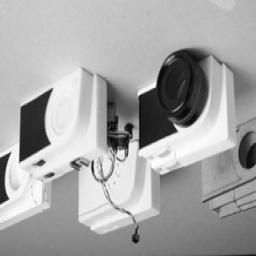

Supplement: Multimedia component 1 [file mmc1.zip › images/not_3d_printed/003467.jpg]

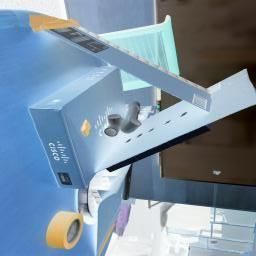

Supplement: Multimedia component 1 [file mmc1.zip › images/not_3d_printed/015211.jpg]

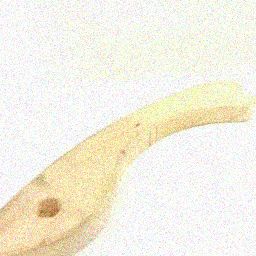

Supplement: Multimedia component 1 [file mmc1.zip › images/not_3d_printed/005002.jpg]

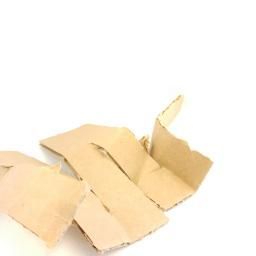

Supplement: Multimedia component 1 [file mmc1.zip › images/not_3d_printed/013660.jpg]

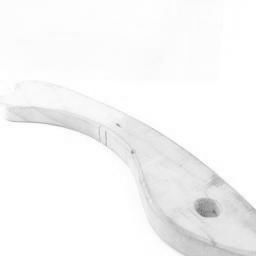

Supplement: Multimedia component 1 [file mmc1.zip › images/not_3d_printed/003473.jpg]

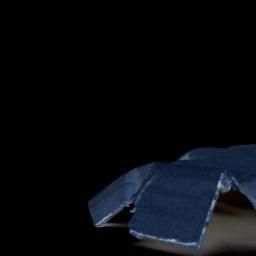

Supplement: Multimedia component 1 [file mmc1.zip › images/not_3d_printed/018735.jpg]

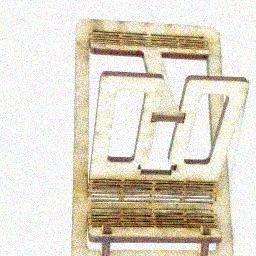

Supplement: Multimedia component 1 [file mmc1.zip › images/not_3d_printed/017406.jpg]

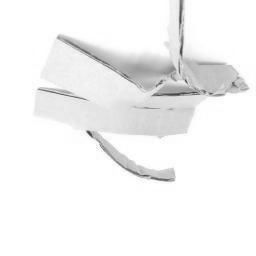

Supplement: Multimedia component 1 [file mmc1.zip › images/not_3d_printed/010369.jpg]

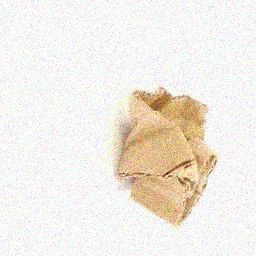

Supplement: Multimedia component 1 [file mmc1.zip › images/not_3d_printed/008526.jpg]

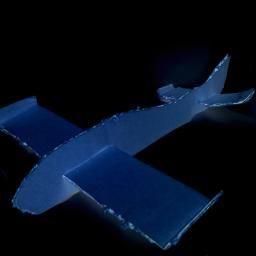

Supplement: Multimedia component 1 [file mmc1.zip › images/not_3d_printed/007615.jpg]

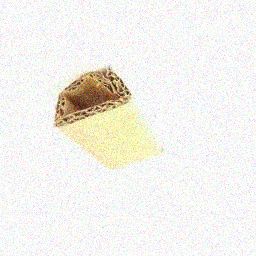

Supplement: Multimedia component 1 [file mmc1.zip › images/not_3d_printed/009638.jpg]

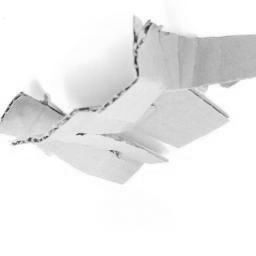

Supplement: Multimedia component 1 [file mmc1.zip › images/not_3d_printed/011077.jpg]

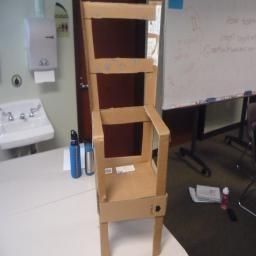

Supplement: Multimedia component 1 [file mmc1.zip › images/not_3d_printed/001264.jpg]

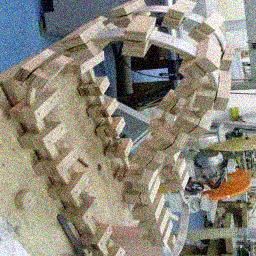

Supplement: Multimedia component 1 [file mmc1.zip › images/not_3d_printed/016718.jpg]

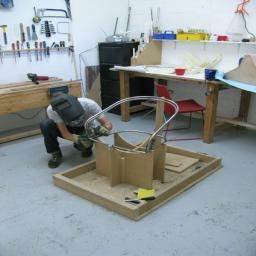

Supplement: Multimedia component 1 [file mmc1.zip › images/not_3d_printed/001502.jpg]

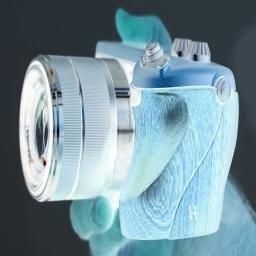

Supplement: Multimedia component 1 [file mmc1.zip › images/not_3d_printed/011711.jpg]

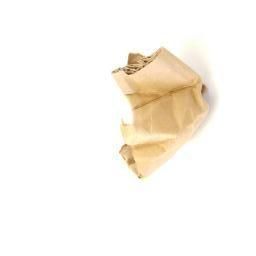

Supplement: Multimedia component 1 [file mmc1.zip › images/not_3d_printed/008240.jpg]

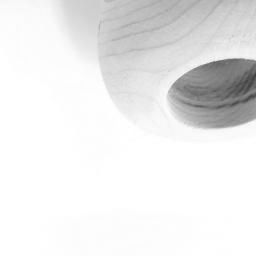

Supplement: Multimedia component 1 [file mmc1.zip › images/not_3d_printed/007173.jpg]

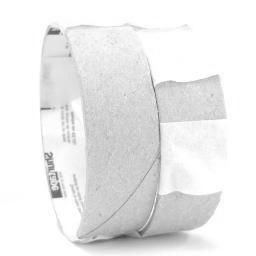

Supplement: Multimedia component 1 [file mmc1.zip › images/not_3d_printed/018053.jpg]

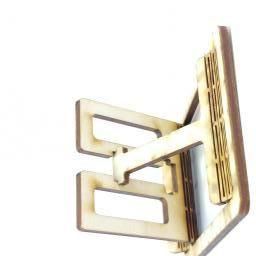

Supplement: Multimedia component 1 [file mmc1.zip › images/not_3d_printed/017360.jpg]

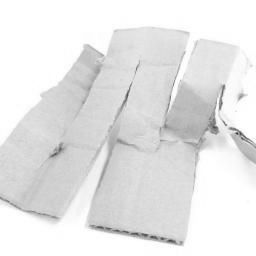

Supplement: Multimedia component 1 [file mmc1.zip › images/not_3d_printed/014669.jpg]

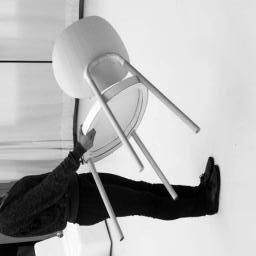

Supplement: Multimedia component 1 [file mmc1.zip › images/not_3d_printed/003315.jpg]

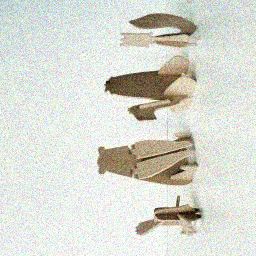

Supplement: Multimedia component 1 [file mmc1.zip › images/not_3d_printed/013106.jpg]

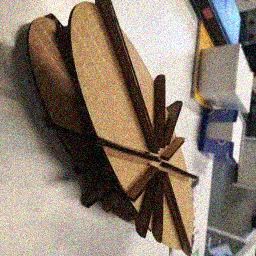

Supplement: Multimedia component 1 [file mmc1.zip › images/not_3d_printed/005764.jpg]

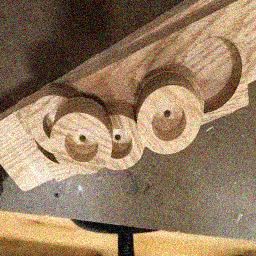

Supplement: Multimedia component 1 [file mmc1.zip › images/not_3d_printed/012218.jpg]

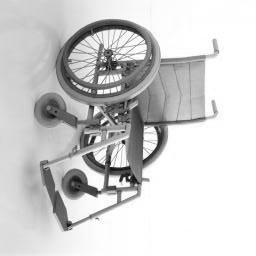

Supplement: Multimedia component 1 [file mmc1.zip › images/not_3d_printed/015577.jpg]

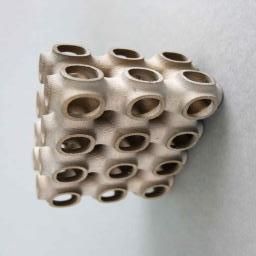

Supplement: Multimedia component 1 [file mmc1.zip › images/not_3d_printed/024972.jpg]

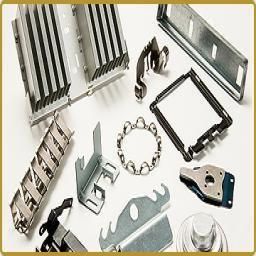

Supplement: Multimedia component 1 [file mmc1.zip › images/not_3d_printed/016056.jpg]

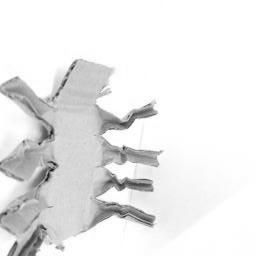

Supplement: Multimedia component 1 [file mmc1.zip › images/not_3d_printed/019365.jpg]

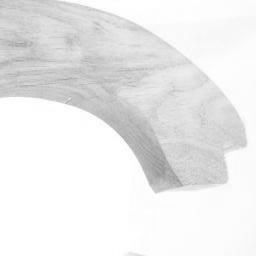

Supplement: Multimedia component 1 [file mmc1.zip › images/not_3d_printed/006245.jpg]

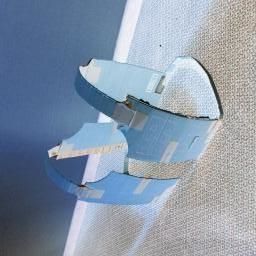

Supplement: Multimedia component 1 [file mmc1.zip › images/not_3d_printed/011739.jpg]

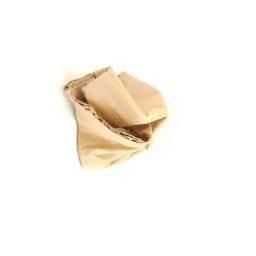

Supplement: Multimedia component 1 [file mmc1.zip › images/not_3d_printed/009176.jpg]

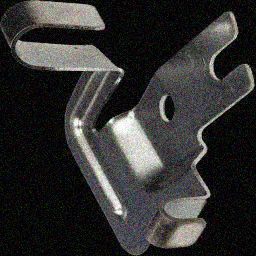

Supplement: Multimedia component 1 [file mmc1.zip › images/not_3d_printed/021822.jpg]

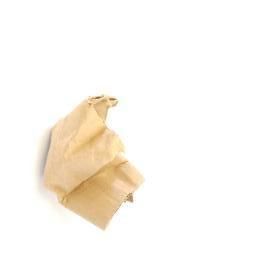

Supplement: Multimedia component 1 [file mmc1.zip › images/not_3d_printed/008268.jpg]

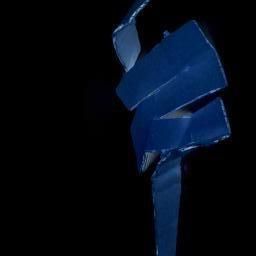

Supplement: Multimedia component 1 [file mmc1.zip › images/not_3d_printed/010427.jpg]

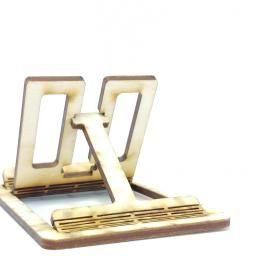

Supplement: Multimedia component 1 [file mmc1.zip › images/not_3d_printed/017348.jpg]

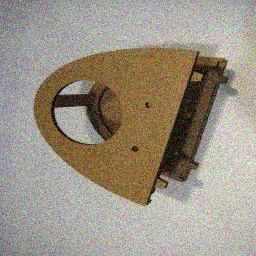

Supplement: Multimedia component 1 [file mmc1.zip › images/not_3d_printed/000634.jpg]

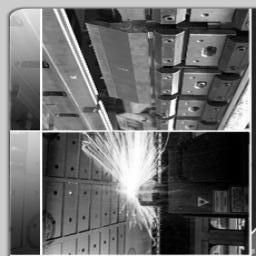

Supplement: Multimedia component 1 [file mmc1.zip › images/not_3d_printed/014641.jpg]

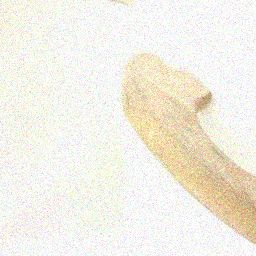

Supplement: Multimedia component 1 [file mmc1.zip › images/not_3d_printed/005994.jpg]

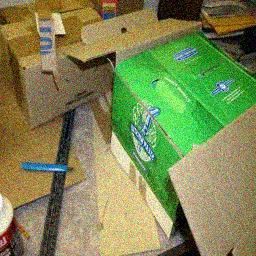

Supplement: Multimedia component 1 [file mmc1.zip › images/not_3d_printed/024782.jpg]

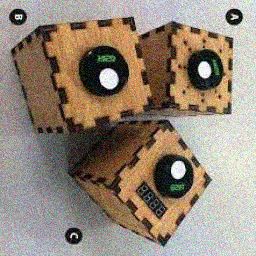

Supplement: Multimedia component 1 [file mmc1.zip › images/not_3d_printed/004452.jpg]

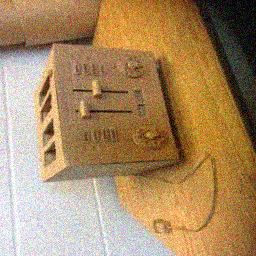

Supplement: Multimedia component 1 [file mmc1.zip › images/not_3d_printed/012230.jpg]

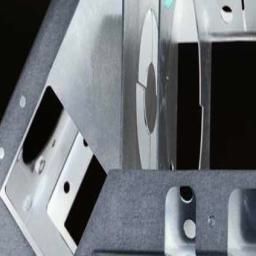

Supplement: Multimedia component 1 [file mmc1.zip › images/not_3d_printed/014899.jpg]

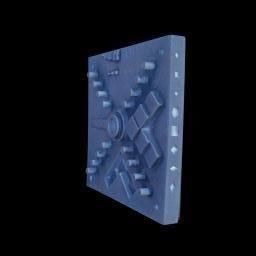

Supplement: Multimedia component 1 [file mmc1.zip › images/not_3d_printed/002023.jpg]

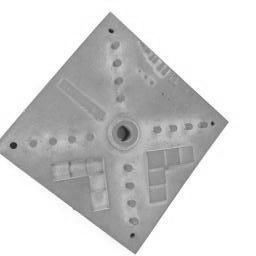

Supplement: Multimedia component 1 [file mmc1.zip › images/not_3d_printed/002745.jpg]

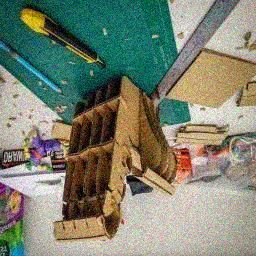

Supplement: Multimedia component 1 [file mmc1.zip › images/not_3d_printed/013890.jpg]

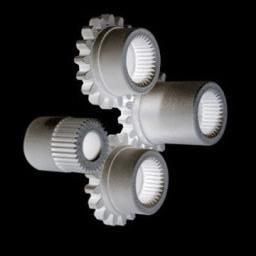

Supplement: Multimedia component 1 [file mmc1.zip › images/not_3d_printed/015239.jpg]

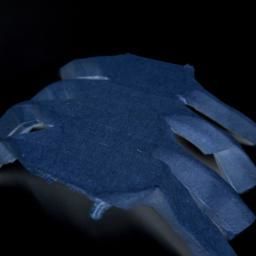

Supplement: Multimedia component 1 [file mmc1.zip › images/not_3d_printed/022495.jpg]

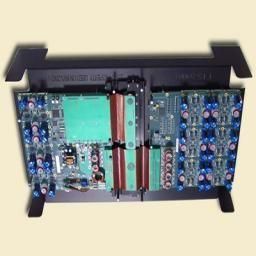

Supplement: Multimedia component 1 [file mmc1.zip › images/not_3d_printed/012556.jpg]

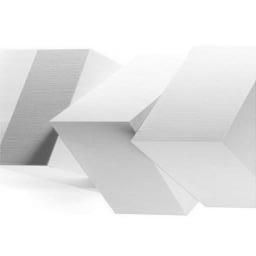

Supplement: Multimedia component 1 [file mmc1.zip › images/not_3d_printed/023953.jpg]

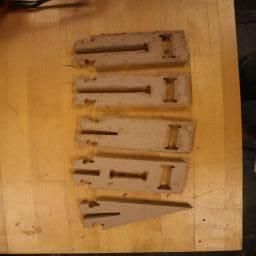

Supplement: Multimedia component 1 [file mmc1.zip › images/not_3d_printed/013648.jpg]

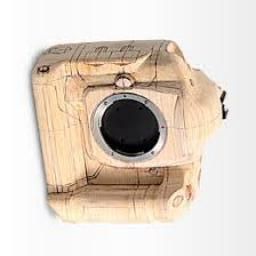

Supplement: Multimedia component 1 [file mmc1.zip › images/not_3d_printed/004334.jpg]

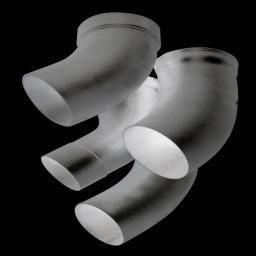

Supplement: Multimedia component 1 [file mmc1.zip › images/not_3d_printed/014127.jpg]

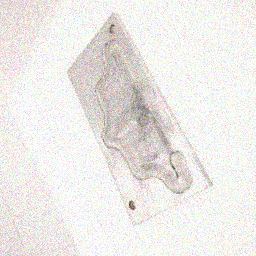

Supplement: Multimedia component 1 [file mmc1.zip › images/not_3d_printed/020282.jpg]

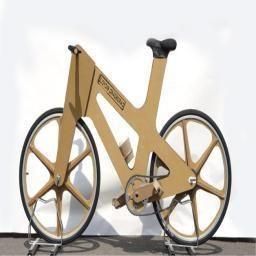

Supplement: Multimedia component 1 [file mmc1.zip › images/not_3d_printed/000152.jpg]

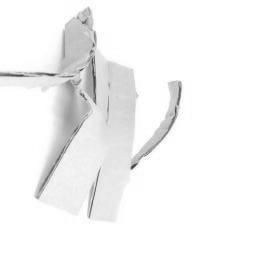

Supplement: Multimedia component 1 [file mmc1.zip › images/not_3d_printed/010341.jpg]

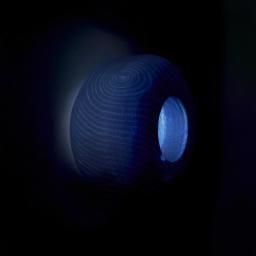

Supplement: Multimedia component 1 [file mmc1.zip › images/not_3d_printed/006523.jpg]

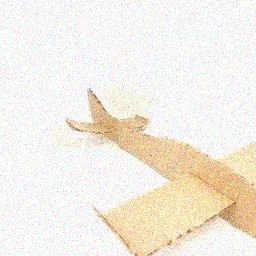

Supplement: Multimedia component 1 [file mmc1.zip › images/not_3d_printed/009610.jpg]

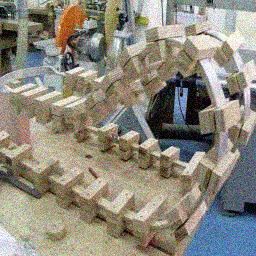

Supplement: Multimedia component 1 [file mmc1.zip › images/not_3d_printed/016730.jpg]

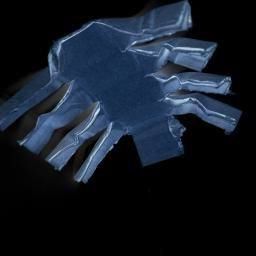

Supplement: Multimedia component 1 [file mmc1.zip › images/not_3d_printed/019403.jpg]

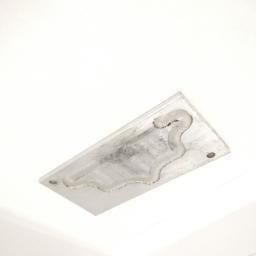

Supplement: Multimedia component 1 [file mmc1.zip › images/not_3d_printed/020296.jpg]

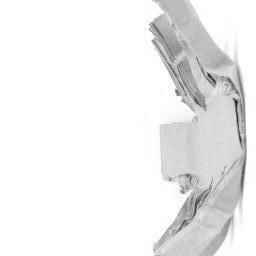

Supplement: Multimedia component 1 [file mmc1.zip › images/not_3d_printed/018709.jpg]

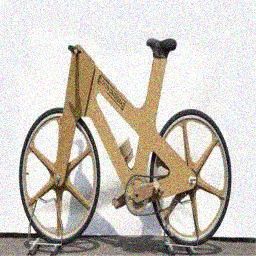

Supplement: Multimedia component 1 [file mmc1.zip › images/not_3d_printed/000146.jpg]

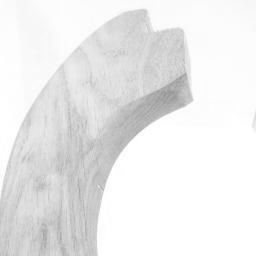

Supplement: Multimedia component 1 [file mmc1.zip › images/not_3d_printed/007629.jpg]

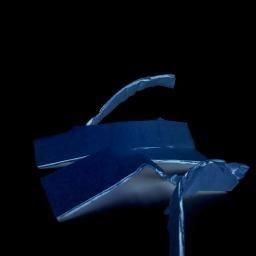

Supplement: Multimedia component 1 [file mmc1.zip › images/not_3d_printed/010355.jpg]

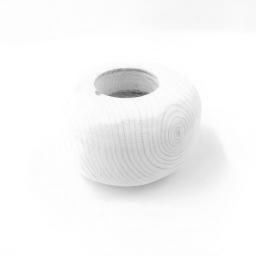

Supplement: Multimedia component 1 [file mmc1.zip › images/not_3d_printed/006537.jpg]

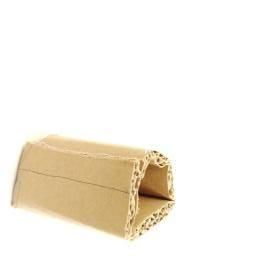

Supplement: Multimedia component 1 [file mmc1.zip › images/not_3d_printed/009604.jpg]

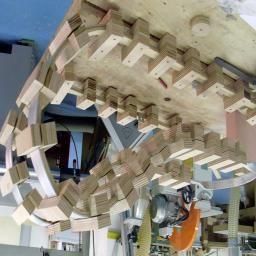

Supplement: Multimedia component 1 [file mmc1.zip › images/not_3d_printed/016724.jpg]

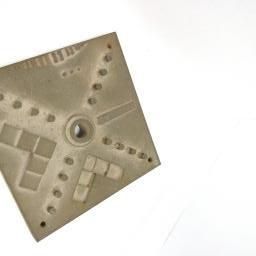

Supplement: Multimedia component 1 [file mmc1.zip › images/not_3d_printed/021188.jpg]

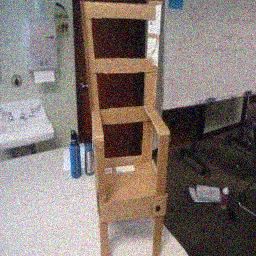

Supplement: Multimedia component 1 [file mmc1.zip › images/not_3d_printed/001258.jpg]

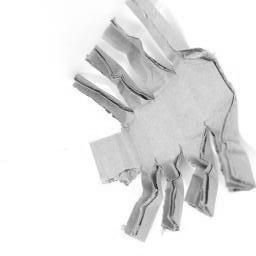

Supplement: Multimedia component 1 [file mmc1.zip › images/not_3d_printed/019417.jpg]

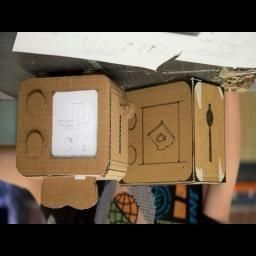

Supplement: Multimedia component 1 [file mmc1.zip › images/not_3d_printed/013884.jpg]

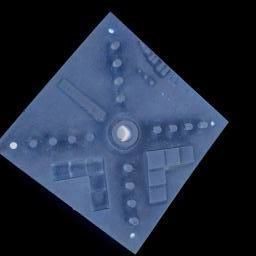

Supplement: Multimedia component 1 [file mmc1.zip › images/not_3d_printed/002751.jpg]

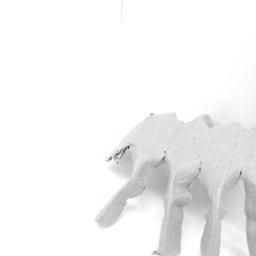

Supplement: Multimedia component 1 [file mmc1.zip › images/not_3d_printed/022481.jpg]

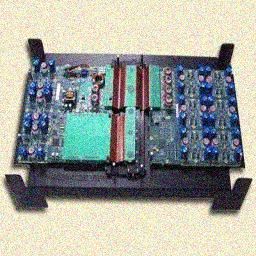

Supplement: Multimedia component 1 [file mmc1.zip › images/not_3d_printed/012542.jpg]
